# Factors associated with emotional exhaustion in healthcare professionals involved in the COVID-19 pandemic: an application of the job demands-resources model

**DOI:** 10.1007/s00420-021-01669-z

**Published:** 2021-03-03

**Authors:** Serena Barello, Rosario Caruso, Lorenzo Palamenghi, Tiziana Nania, Federica Dellafiore, Loris Bonetti, Andrea Silenzi, Claudia Marotta, Guendalina Graffigna

**Affiliations:** 1 EngageMinds HUB, Consumer, Food and Health Engagement Research Center, Università Cattolica del Sacro Cuore, L.go Gemelli 1, 20123 Milan, Italy; 2 Department of Psychology, Università Cattolica del Sacro Cuore, L.go Gemelli 1, 20123 Milan, Italy; 3 Faculty of Psychology, Università Cattolica del Sacro Cuore, L.go Gemelli 1, 20123 Milan, Italy; 4 Health Professions Research and Development Unit, IRCCS Policlinico San Donato, San Donato Milanese, MI Italy; 5 Faculty of Agriculture, Food and Environmental Sciences, Università Cattolica del Sacro Cuore, via Milano 24, 26100 Cremona, Italy; 6 Ente Ospedaliero Cantonale and Research and Development Unit of Oncology, Nursing Research Centre, Oncology Institute of Southern Switzerland (IOSI), 6500 Bellinzona, Switzerland; 7 Ministry of Health, Rome, Italy; 8 Center for Leadership in Medicine Research and Studies, Università Cattolica del Sacro Cuore, Rome, Italy

**Keywords:** COVID-19, Healthcare professionals, Burnout, Job demands-resources model, Work engagement, Patient engagement

## Abstract

**Purpose:**

The purpose of the present cross-sectional study is to investigate the role of perceived COVID-19-related organizational demands and threats in predicting emotional exhaustion, and the role of organizational support in reducing the negative influence of perceived COVID-19 work-related stressors on burnout. Moreover, the present study aims to add to the understanding of the role of personal resources in the Job Demands-Resources model (JD-R) by examining whether personal resources—such as the professionals’ orientation towards patient engagement—may also strengthen the impact of job resources and mitigate the impact of job demands.

**Methods:**

This cross-sectional study involved 532 healthcare professionals working during the COVID-19 pandemic in Italy. It adopted the Job-Demands-Resource Model to study the determinants of professional’s burnout. An integrative model describing how increasing job demands experienced by this specific population are related to burnout and in particular to emotional exhaustion symptoms was developed.

**Results:**

The results of the logistic regression models provided strong support for the proposed model, as both Job Demands and Resources are significant predictors (OR = 2.359 and 0.563 respectively, with *p* < 0.001). Moreover, healthcare professionals’ orientation towards patient engagement appears as a significant moderator of this relationship, as it reduces Demands’ effect (OR = 1.188) and increases Resources’ effect (OR = 0.501).

**Conclusions:**

These findings integrate previous findings on the JD-R Model and suggest the relevance of personal resources and of relational factors in affecting professionals’ experience of burnout.

## Introduction

The COVID-19 spread caused, in a few weeks, excessive hospital overload, a high shortage of healthcare resources, and an additional workload for professionals. Hospitals had to rapidly reconfigure clinical spaces and restructure work teams to address the surge of patients diagnosed with COVID-19 (Miller et al. 2020). Many healthcare workers have, therefore, been often redeployed to settings outside their usual clinical specialty and/or experience, often working extra shifts and longer hours to meet the high volume of patient demand. Moreover, the COVID-19 pandemic has required professionals’ to revise their relational models to interact with patients due to the recommendation to prevent the contagion spread. In addition to that, as SARS-CoV-2 is highly infectious, healthcare workers have been at increased risk for acquiring and potentially transmitting COVID-19 to patients, co-workers, and family/friends. Therefore, the interplays among these stressful triggers made healthcare workers face unprecedented amounts of psychological distress between professional and personal life (Fabiana et al. 2018; Barello et al. 2020a, b), in addition to anxiety and depression (Chen et al. 2020a).

The large amount of research conducted after the pandemic outbreak unanimously demonstrated that healthcare professionals have been observed in this period to experience serious psychological problems and to be at risk in terms of mental health (Dewey et al. 2020). Among the common mental effects of the pandemic are anxiety, panic, depression, anger, confusion, ambivalence and financial stress (Kang et al. 2020). Healthcare workers were observed to experience similar problems during previous pandemics (Barello et al. 2020a). Depression, anxiety and post-traumatic stress disorder are the most common psychological disorders that were reported particularly in healthcare professionals during the 2003 SARS and 2014 Ebola virus pandemics (Chan and Chan 2004; Chua et al. 2004). Studies have also shown that healthcare professionals are considerably more worried about catching the infection during a pandemic. Exposure to COVID-19 patients raises anxiety and fear of virus infection. As a result, levels of stress, depression and anxiety rise in healthcare workers and they might become traumatized (Tan et al. 2020). According to Cullen et al. (2020), particularly those working in public health, primary care, emergency service and intensive care are at the risk of developing psychological symptoms. Nonetheless, the identification of processes through which work-related and personal characteristics affected healthcare professionals’ wellbeing during the COVID-19 pandemic has received still little research attention (Chen et al. 2020b; Lai et al. 2020; The Lancet 2020). Only few studies conducted have focused on job-related factors and have revealed that healthcare workers are exposed to work overload, isolation and discrimination, and therefore they experience exhaustion, fear, affective disorders and sleep problems (Gavin et al. 2020; Lai et al. 2020). Thus, it is critical to identify protective factors—both personal and work-related—to prevent the onset of burnout symptoms.

Literature provides various psychological models that explain how stressful situations impact on job performances. For our study, we have chosen to test the Job Demands-Resources (JD-R) Model (Bakker and Demerouti 2008). This model assumes that whereas every work may have its own specific risk factors associated with job stress and burnout, these factors can be classified into two main categories: job demands and job resources. Job demands are organizational psycho-social aspects of work that require cognitive and emotional efforts, generally associated with social or psychological costs. On the other hand, job resources are physical, psycho-social, and organizational aspects of work, which facilitate the achievement of work objectives, professional growth, personal development, and reduce job demands and the -psychological or physiological costs associated with them (Bakker and Demerouti 2007).

Recently, there has been a growing interest also in the role of personal resources in the relationships between job characteristics and professionals’ mental health. Personal resources (e.g., hope, optimism, resilience, and empathy) may herein be conceptualized as individuals’ strengths or characteristics that might contribute to individuals’ optimal functioning (Youssef and Luthans 2007). Different studies have found empirical evidence that personal resources act as buffers for job demands (Xanthopoulou et al. 2007a). For instance, research showed that professionals’ resilience is a significant predictor of the psychological wellbeing of healthcare workers (Arrogante and Aparicio-Zaldivar 2017; Guo et al. 2018); moreover, other studies suggested a negative relationship between burnout and empathy amongst healthcare staff (i.e., high burnout–low empathy) (Wilkinson et al. 2017). Evidence from different healthcare settings suggested that the quality of patient–provider relationships, and in particular of a relationship oriented to actively involve patients in their healthcare pathway, are related to both patients and providers’ positive outcomes (Carlsen and Aakvik 2006; Ratanawongsa et al. 2008). Particularly, poor or insufficient professionals’ orientation toward the psycho-social needs and engagement of patients has been found to contribute to physicians’ burnout (Travado et al. 2005): interestingly, healthcare workers with lower attitudes towards the engagement their patients’ also reported high levels of burnout, namely emotional exhaustion and depersonalization (Arora 2003). Thus, literature suggests that the strains of an asymmetric relationship (i.e., not oriented to the active engagement of patients in the clinical path) between healthcare professionals and their patients may eventually deplete clinicians' emotional resources and initiate the burnout syndrome (Bakker et al. 2000).

Therefore, the purpose of the present study is to investigate the role of perceived COVID-19-related organizational demands and threats in predicting emotional exhaustion, and the role of organizational support in reducing the negative influence of perceived COVID-19 work-related stressors on burnout. Moreover, the present study aims to add to the understanding of the role of personal resources in the JD-R model by examining whether personal resources may also strengthen the impact of job resources and mitigate the impact of job demands.

In line with earlier research on occupational stress and burnout (Lee and Ashforth 1996), our model predicts that healthcare professionals’ exposure to COVID-19-specific job demands leads to feelings of emotional exhaustion, while job and personal resources are expected to reduce emotional exhaustion symptoms (hypothesis 1).

Our second hypothesis states that professionals’ orientation towards patient engagement would mitigate the negative effect of job demands on emotional exhaustion experience and enhance the buffering effect of job and personal resources (hypothesis 2).

As far as we know, this specific pattern of relationships has not been tested simultaneously in earlier studies.

## Methods

### Study design

This research is a cross-sectional study, which is part of a larger research project (named “C.O.P.E.” study: Covid19-related Outcomes of health Professionals during the Epidemic). A web-based survey was created in March 2020, during phase one of the Covid-19 outbreak in Italy, and was administered in April and May, which were the months where the Covid-19 outbreak reached its peak in Italy, using the online platform Qualtrics^®^.

### Survey development and measurements

The adopted approach to develop the web-based survey was consistent with previously published recommendations for conduction survey research (Schleyer and Forrest 2000). Initially, the authors delineated the sections of the investigation, based on an initial literature search performed to generate the hypotheses of this study. Accordingly, the sections of the web-survey were (a) the form for collecting the socio-demographic and professional characteristics, (b) the measures of job demands and resources, (c) the orientation towards patient engagement; (d) the empathy, (e) the resilience, and (f) the emotional exhaustion.

The socio-demographic and professional characteristics collected were: sex (male, female), age (years), nationality (Italian, other), marital status (unmarried, married, or in a relationship), occupation (physician, nurse, and other), region, specific provenience to “red zones” during the COVID-19 outbreak (yes, no), being a caregiver of a relative (yes, no). As for occupation, under the label “other” fall all those healthcare workers who are not either physician or nurses (the so called “allied health professionals”): technicians, social workers, and other supporting professionals. While labeling all those different professions under the same category results in a loss of information; currently in Italy, there are about 20 different recognized non-medical health professions: as this is not a core variable in our study, we were not interested in such a level of detail.

The measures of job demands, job resources, and professionals’ orientation towards patient engagement were developed ad hoc, as no reliable and valid tools were found to fit with the characteristics derived from the COVID-19 pandemic. Although the development of ad hoc measures may have reduced the measurements’ reliability and validity compared to the use of already-existing measures, this approach is consistent with the recommendations for developing studies using the JD-R model as a theoretical framework (Bakker and Demerouti 2008), and, moreover, allows for a more focused measurement of the constructs of interest and for the use of items more contextualized in the current pandemic.

Participants were asked to rate their agreement to a series of statements on a six-point Likert scale: nine items regarding job demands, six items for job resources, and six items for the orientation towards patient engagement (please, see Appendix A for a full list of the items).

Empathy was measured using an adapted version of the Jefferson Scale of Empathy (JSE) (Kane et al. 2007). The JSE is a valid and reliable self-report scale, which encompasses 20 items to measure empathy in healthcare contexts. The JSE has to be computed to score its three domains: Perspective Taking (ten items), Compassionate Care (eight items), and Standing in the Patient’s Shoes (two items). Each item is answered on a seven-point Likert scale.

The resilience was measured using the Brief Resilience Scale (BRS) (Smith et al. 2008). The BRS purposes of evaluating the individual’s ability to recover from stressful events; it encompasses six items. The BRS has a 5-point Likert response: given the scale one-factor structure, the higher the score, the greater the resilience.

The sub-scale of the Maslach Burnout Inventory-Italian version (MBI) regarding emotional exhaustion was used and adapted for the specific purposes of this study (Maslach et al. 1997). This sub-scale included nine items, and it previously showed high internal consistency (Worley et al. 2008). Each of the nine items asks healthcare professionals to describe their feelings on a 7-points-Likert scale, ranging from never having those feelings to having those feelings a few times a week.

### Sampling and procedure

A snowball sampling approach was used to enroll Italian healthcare providers, such as physicians, nurses, or other professionals. Two sampling managers, which were identified among authors, took care of sampling via snowballing by recruiting participants either via email/social network invitations, or by asking medical directors of various healthcare facilities to involve the healthcare professionals working there; finally, specific referents from the regions of Italy with a higher spread of Covid-19 infection were also involved in the dissemination of the survey using his/her own network.

Each professional involved that answered the survey was asked to invite other eligible future volunteers from their network of contacts.

The invitations contained all the relevant information regarding the study and its aims, as well as an online link to access to the questions. Once the participants accessed the survey, a self-assessment check of eligibility was asked before proceeding with the questions. The eligibility check aimed to describe the socio-demographic characteristics of the sample, where the healthcare professionals had to state their specific profession and educational background.

### Data analysis

Data analysis was performed in four main phases: (1) initial data check, descriptive statistics, and correlational analysis; (2) dimensionality of the ad hoc developed items and included self-report measurements to support the scoring procedure and decrease measurement bias; (3) testing the effects of the protective and risk factors on emotional exhaustion; (4) testing the interaction (moderation) of the orientation towards patient engagement in the relationships from the protective and risk factors to the emotional exhaustion.

First, the data were assessed for missing information, errors, or outliers using the frequency check. In this phase, we employed descriptive statistics (mean, standard deviation [SD] and frequency) to summarize the responders’ characteristics, assessing the skewness, and kurtosis of the items. This preliminary check was relevant to choosing the best confirmatory factor analysis (CFA) estimator for the subsequent phase of analysis. A bivariate analysis was initially performed to explore the relationships among the collected variables.

In the second phase, we performed three separate CFAs, one for each developed scale, for corroborating the one-factor structure of the items included to measure job demands, job resources, and orientation towards patient engagement. This approach is consistent with the previous research using multi-scales (Riegel et al., 2004). As some items showed skewness higher than |1.0|, we used the Maximum Likelihood Robust (MLR) estimator determining parameters for each model. As per the evaluation of the goodness of fit, we considered the Comparative Fit Index (CFI), Tucker and Lewis Index (TLI), Root Mean Square Error of Approximation (RMSEA), and Standardized Root Mean Square Residual (SRMR). The CFI and TLI values of ≥ 0.90 indicate a well-fitting model, RMSEA with values ≤ 0.08 indicates as an adequate-fitting model, SRMR with values ≤ 0.08 indicates a good fit for the employed model. We further considered as fit indices the chi-square test (*χ*^2^) and the ratio *χ*^2^/degrees of freedom. Factor loadings were considered as adequate when higher than |0.30|. We further examined the internal consistency of each scale by computing the Cronbach’s alpha coefficient. Once confirmed the adequacy of the dimensionality of the measures of job demands, job resources, and the orientation towards patient engagement (ad hoc developed items), the scoring for each domain was performed using the mean of the items kept by each domain.

In the third phase, a logistic regression (LR) model with the estimation of the unknown parameters through the maximum likelihood was used to assess the associations between the protective-risk factors (covariates) on emotional exhaustion within the generalized linear models’ framework. Accordingly, the outcome (emotional exhaustion) was dichotomized following the official Italian cut-offs for healthcare workers (Sirigatti and Stefanile 1992): scores ≥ 24 indicate high emotional exhaustion. We further included as covariate the variable indicating whether or not the enrolled healthcare worker had to act as a caregiver for a relative, as this variable resulted in being correlated with the emotional exhaustion in the correlational analysis. Covariates were simultaneously included in the model. The goodness of fit was assessed using the Hosmer–Lemeshow Test (non-significant *P* indicate a good fit), and the Nagelkerke’s pseudo-*R*^2^. The data were reported as adjusted odds ratio (OR) and 95% confidence interval (CI) with model–robust sandwich standard error estimates.

In the fourth phase, to evaluate whether the orientation towards patient engagement modified the association between any covariates and emotional exhaustion, we run the LR model including the interactions between the orientation towards patient engagement and the model covariates, maintaining the same strategy for evaluating the goodness of fit and for the reporting of the results. Overall, for interferential analyses, we set a significance level of 0.05, using IBM SPSS 22 (SPSS, Inc., Chicago, IL, USA) and Mplus V8.1.

## Results

### Sample characteristics

Overall, 744 healthcare professionals were reached by the snowball sampling and 532 of them agreed to be involved (response rate = 71.5%). As described in Table 1, the participants were mainly females (*n* = 399; 75%), Italians (*n* = 512; 96.2%), married (*n* = 312; 58.6%), nurses (*n* = 327; 61.5%), from the Lombardy region (*n* = 323; 60.7%), and from “red zones” during COVID-19 outbreak (*n* = 417; 72.4%). Only 84 participants reported to act as caregiver for a relative (15.8%). The mean (SD) age of the responders was 41.06 (11.16) years.

**Table 1:** Characteristics of the sample (*N* = 532)

	*N*	%
Sex		
Male	133	25
Female	399	75
Age		
Years (mean; SD)	41.06 (11.16)	
Nationality		
Italian	512	96.2
Other	20	4.8
Marital status		
Unmarried and not in a relationship	220	41.4
Married or in a relationship	312	58.6
Occupation		
Physician	106	19.9
Nurses	327	61.5
Other	99	18.6
Region of Italy		
Piedmont	11	2.1
Lombardy	323	60.7
Veneto	49	9.2
Emilia Romagna	34	6.4
Other	115	21.6
“Red zone” during Covid-19 Outbreak		
Yes	417	72.4
No	115	21.6
The respondent is a caregiver		
Yes	84	15.8
No	448	84.2

### The dimensionality of the ad hoc developed scales

We tested a confirmatory model to evaluate whether the one-factor structure, theorized to score the six items for job resources, was an adequate solution to explain data. The model (*χ*^2^_(31)_ = 107.061, *p* < 0.001; *χ*^2^/df = 3.4; RMSEA = 0.067; 90% CI [0.059–0.082]; CFI = 0.904; TLI = 0.900; and SRMR = 0.049) was satisfactory (standardized factor loadings ranged between 0.595 and 0.821; Cronbach’s alfa = 0.865). Likewise, the confirmatory model (*χ*^2^_(15)_ = 47.081, p < 0.001; *χ*^2^/df = 3.1; RMSEA = 0.057; 90% CI [0.009–0.088]; CFI = 0.989; TLI = 0.967; and SRMR = 0.023) used to assess the adequacy of the one-factor solution for the domain of job demands was satisfactory (9 items; standardized factor loadings ranged between 0.662 and 0.762; Cronbach’s alfa = 0.914). Finally, the model performed to confirm the one-factor structure of the orientation towards patient engagement (6 items) was satisfactory as well (*χ*^2^_(9)_ = 42.563, *p* < 0.0001; *χ*^2^/df = 4.7; RMSEA = 0.079; 90% CI [0.051–0.098]; CFI = 0.899; TLI = 0.867; and SRMR = 0.056), also showing that standardized factor loadings ranged between 0.402 and 0.798; Cronbach’s alfa = 0.841).

### Scores of emotional exhaustions and its theoretical risk and protective factors

As described in Table 2, emotional exhaustion showed a mean score of 22.2 (12.2); more precisely, several participants had a score indicating high emotional exhaustion (*n* = 183; 33.6%). Among the ad hoc developed measures, the mean (SD) score of job demands was 4.3 (0.8), the mean (SD) score of job resources was 3.9 (1.2), and the mean (SD) score of the orientation to patient engagement was 4.8 (0.7). Among the subscales of the JSE, the mean (SD) scores of standing in the patient’s shoes, compassionate care, and perspective-taking were 5.5 (0.6), 5.3 (0.6), 4.8 (0.8), respectively. The mean (SD) score of the BRS was 3.3 (0.8).

**Table 2:** Mean scores of the measured constructs

JD Overall	Mean	St. Dev
Ad hoc developed measures		
Job demands	4.3	0.8
Job resources	3.9	1.2
Orientation to patient engagement	4.8	0.7
Empathy		
Compassionate care	5.3	0.6
Standing in the patient’s shoes	5.5	0.6
Perspective taking	4.8	0.6
Resilience		
Score	3.3	0.8
Emotional exhaustion		
Score	22.2	12.2
Low emotional exhaustion (*n*; %)	211	38.8
Moderate emotional exhaustion (*n*; %)	150	27.6
High emotional exhaustion (*n*; %)	183	33.6

The bivariate analysis showed no significant correlations between socio-demographic and professional characteristics (described in Table 1) with emotional exhaustion. Conversely, the correlations between emotional exhaustion and theoretical protective factors were negative: emotional exhaustion with job resources (*r* = − 0.321; *p* < 0.001), orientation to patient engagement (*r* =− 0.181; *p*  < 0.001), empathy [compassionate care (*r* = − 0.137; *p* < 0.001), standing in patient’s shoes (r = − 0.140; *p* < 0.001), perspective taking (*r* = − 0.082; *p* < 0.058), and resilience (*r* = − 0.204; *p* < 0.001)]. The correlation between emotional exhaustion and job demands (theoretical risk factor) was positive (*r* = 0.262; *p* < 0.001).

### Determinants of emotional exhaustion: protective and risk factors

As described in Table 3, each additional point of the score of orientation towards patient engagement decreases by roughly 27% the odds of high emotional exhaustion (OR = 0.731; 95%CI  [0.521 − 1.001]; *p* = 0.0501). Likewise, each additional point of job resources decreases by roughly 44% the odds of high emotional exhaustion (OR = 0.563; 95%CI [0.461 − 0.687]; *p* < 0.001). Each additional point of resilience decreases by roughly 27% the odds of high emotional exhaustion (OR = 0.727; 95%CI  [0.558 − 0.947]; *p* < 0.001). Conversely, each additional point of job demands increases by roughly 2.4 times the odds of high emotional exhaustion (OR = 2.359; 95%CI  [1.673 − 3.325]; *p* < 0.001). No significant associations were found regarding the actions of empathy sub-scales on emotional exhaustion.

**Table 3:** Effects of protective and risk factors on emotional exhaustion

	OR	95%CI		*p*
Covariates				
Job demands	2.359	1.673	3.325	< 0.001
Job resources	0.563	0.461	0.687	< 0.001
Orientation to patient engagement	0.731	0.521	1.001	0.0501
Perspective taking	0.975	0.761	1.093	0.098
Compassionate care	0.705	0.420	1.183	0.185
Standing in the patient’s shoes	0.810	0.504	1.302	0.384
Resilience	0.727	0.558	0.947	0.018
Model				
Test Hosmer and Lemeshow (*p* value)	0.341			
Pseudo-*R*^2^ Nagelkerke	0.283			

Outcome = High emotional exhaustion versus moderate/low levels

### Interaction of orientation towards patient engagement in the associations between protective and risk factors with emotional exhaustion

As described in Table 4, the interaction of orientation to patient engagement with job resources decreases by roughly 50% the odds of high emotional exhaustion (OR = 0.501; 95%CI  [0.411 –0.887]; *p* < 0.001), enhancing the exclusive contribution of job resources described in Table 3. Likewise, the interaction of orientation to patient engagement with resilience decreases by roughly the odds of high emotional exhaustion (OR = 0.688; 95%CI  [0.559–0.875]; *p* < 0.001), enhancing the exclusive contribution of resilience on emotional exhaustion, described in Table 3. The interaction of orientation to patient engagement with job demands increases by 18% the odds of high emotional exhaustion (OR = 1.188; 95%CI  [1.120 –1.265]; *p* < 0.001), decreasing the exclusive harmful effect of job demands on emotional exhaustion (Table 3). Overall, adding in the model, the interaction of orientation towards patient engagement with the covariates mitigated the risk factors of emotional exhaustion (job demands), enhancing the effects of its protective factors (job resources and resilience).

**Table 4:** Interaction of orientation towards patient engagement in the associations between protective and risk factors with emotional exhaustion

	OR	95%CI		*p*
Covariates * orientation to patient engagement				
Job demands	1.188	1.120	1.265	< 0.001
Job resources	0.501	0.411	0.887	< 0.001
Perspective Taking	0.963	0.788	1.035	0.081
Compassionate Care	0.913	0.884	1.033	0.163
Standing in the Patient’s Shoes	0.961	0.865	1.074	0.312
Resilience	0.688	0.559	0.875	< 0.001
Model				
Test Hosmer and Lemeshow (*p* value)	0.602			
Pseudo-*R*^2^ Nagelkerke	0.288			

Outcome = High emotional exhaustion versus moderate/low levels

## Discussion

Our first results from the factorial analyses show that the developed scales to measure Covid-19 related job demands and resources, as well as the orientation towards patient engagement, are adequate and unidimensional, with mostly adequate factor loadings.

Descriptive analyses also show that a significant amount of participants (about one out of three) had a high level of emotional exhaustion. This result is in line with the previous literature on the impact of the COVID-19 and other pandemic emergencies on healthcare professional mental health (Barello et al. 2020a; Braquehais et al. 2020; Giusti et al. 2020; Kulkarni et al. 2020; Özdemir and Kerse 2020). This is particularly worrying because previous studies have demonstrated that this symptom is associated with a decrease in work performance due to negative behaviors towards work (Wright and Cropanzano 1998). In particular, in health workers exposed to traumatic situations, the presence of emotional exhaustion has been related with a reduction in the ability to apply coping strategies (Bittner et al. 2011) or negative attitudes towards work. In addition, the symptoms of exhaustion, related to anxiety, depression, insomnia (Luceño-Moreno et al. 2020; Zhang et al. 2020; Barello et al. 2020b), or other symptoms related to physical pathologies (e.g., cardiovascular problems) (Melamed et al. 2006), can lead to intention to leave the workplace (Labrague and de los Santos 2020), which would cause high costs for the healthcare system.

**Fig. 1:**
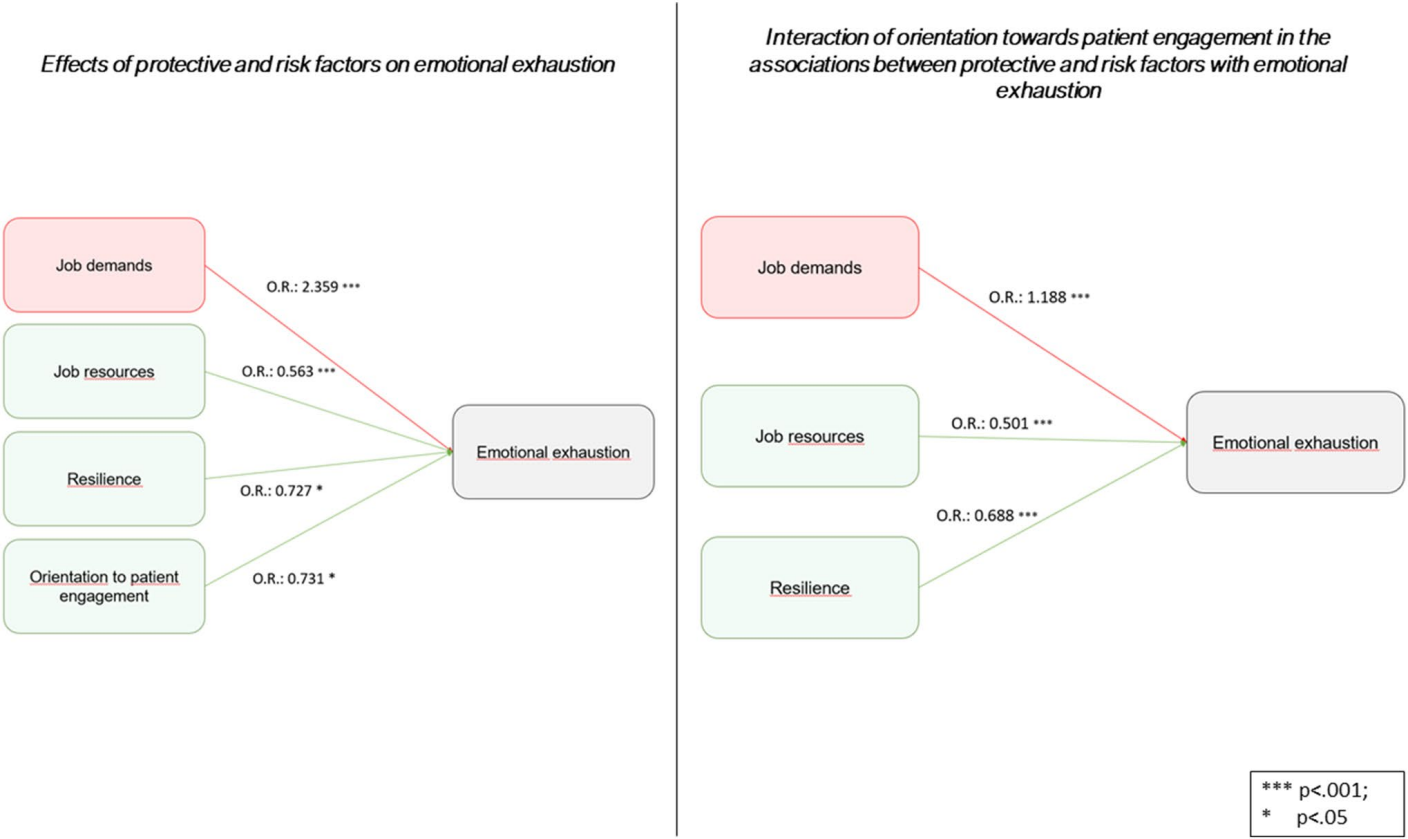
Risk and protective factors for emotional exhaustion and the interaction with professionals’ orientation towards patient engagement

However, the main contribution of this study is that our results contribute to offer a further empirical base to demonstrate theoretical assumptions which are reflective of the JD-R model, and can be used to integrate and expand previous research on work-related stress in healthcare settings. The results show that, as expected and consistent with previous literature, the exposure to job demands leads to feelings of emotional exhaustion, while workplace and personal resources act as a protective factor (Le Blanc et al. 2001; Xanthopoulou et al. 2007b). Moreover, our study shows that professionals’ orientation towards patient engagement has two leading roles: on one side, it acts as a protective factor of emotional exhaustion; on the other side, it significantly mitigates the detrimental effect of demands, also empowering the beneficial effect of resources. This result confirms previous studies linking physician burnout with physician self-reported patient engagement attitudes and behaviors (such as confidence in communication, empathy, or perceived reciprocity in the patient–physician relationship) (Linzer et al. 2001; Paasche-Orlow and Roter 2003; Goehring et al. 2005; Shanafelt et al. 2005; Ratanawongsa et al. 2008) (see Fig. 1).

The present study, then, not only confirms the importance of the JD-R model in a new setting but it is consistent with other research relating to the importance of personal resources in the promotion of health and well-being with implications for the refinement of the JD-R model by specifying the function of personal resources within the framework (Xanthopoulou et al. 2007a; Tremblay and Messervey 2011). Indeed, although longitudinal studies are required to further corroborate this claim, the possible importance of personal resources, as compared to job resources, in regards to the model's dual processes are certainly an interesting novel line of research for future studies. In consideration of all the facts about the current working life of healthcare professionals involved in the COVID-19 emergency, it becomes particularly important to find personal-related stress resistance resources that may modify the well-documented stressor–strain relationship. In the recent research, it has become clear that the tendency to perceive job demands as stressful depends also on the personal resources of the individuals (Mäkikangas and Kinnunen 2003). In our perspective, our findings may have important theoretical and practical implications, especially for frontline healthcare professionals working during the COVID-19 pandemic and healthcare managers.

Indeed, intervention programs aimed at preventing or reducing burnout with its detrimental aspects (e.g., emotional exhaustion) among healthcare professionals may focus upon the proposed model of determinants of the work-related stress. Focusing on the right factors is necessary to promote the general wellbeing of healthcare professionals and, in particular, given the central role of professional orientation to patient engagement in preventing the onset of burnout symptoms, specific dedicated training for healthcare workers aimed at improving their relational attitude and communication skills could be pivotal. As such, these dedicated training focused on fostering professional orientation to patient engagement should be close to daily practice and real-world cases. Of course, besides that, it would be of great relevance to implement job redesign strategies with a careful analysis of healthcare professionals’ daily tasks during the emergency to collect insight into the aspects of their work that are particularly demanding. A rescheduling of the working program and constant supervisors’ support may, in turn, lower the workload and reduce the work pressure. Second, healthcare organizations may consider a reduction of the caseload or a reallocation of tasks related to patient contacts to reduce demanding contacts with patients. In addition, shift-work systems may be optimized to meet the rest needs of clinicians. Such interventions may prevent or reduce feelings of exhaustion among them.

Regarding job resources, which act as protective factors for burnout, according to our results and consistent with job enrichment approaches, it appears crucial to increase healthcare professionals’ participation in decision making regarding their own workload organization (Deckard et al. 1994; Van Bogaert et al. 2009).

Moreover, these interventions may be part of a broader development strategy of health care organizations, aimed at the promotion of a healthy and productive working environment for healthcare professionals, even when occurring healthcare crisis.

## Limitations

The limitations of this study clearly must be noted. First, the analyses in the current study are based on cross-sectional data and, thus, do not confirm causality. In addition, more complex forms of non-recursive linkages could not be examined, as per the cross-sectional nature of the collected data. Second, the present study is based on the self-reports, as per the majority of burnout and stress studies. Self-report data might be contaminated by respondents’ bias. Third, the sample was not representative of the Italian healthcare workers population, and the long questionnaires may have reduced the response rate. Further studies are warranted to test the results of a broader population and to explore the tested model adding socio-demographic variables (i.e., gender, age). Fourth, although our measure of orientation towards patient engagement has been conceptually rooted and its internal consistency was acceptable, it would be necessary further validation studies to confirm its reliability and construct validity. Moreover, the discovery of the crucial role of the professionals’ orientation towards patient engagement suggests the need to deepen the role of relational factors in the JD-R model. Longitudinal studies will help elucidate which factors are associated with a higher risk of developing long-lasting negative psychological effects of the pandemic on healthcare professional. Furthermore, qualitative studies may contribute to understanding the influence of individual and social narratives in HPs’ burnout and distress.

## Concluding remarks

Our results uncover some of the antecedents of burnout among healthcare professionals in facing the challenging situation of the COVID-19 pandemic. Based on our results and acknowledging the high detected rates of professionals with high emotional exhaustion, we suggest the implementation of specific intervention programs, training, and assessments for the healthcare workers being employed during the COVID-19 emergency. As well as emotional exhaustion reduces the commitment to the profession and leads to work disengagement and low patient satisfaction, we think that taking care of health professionals’ mental health in this emergency is an urgent public health issue to be addressed by fostering the organizational resources and by mitigating the organizational demands. Moreover, the discovery of the protective value of professionals’ attitudes towards partnering with patients supports the idea that social capital in the workplace is a resource, as it helps people to cope with stress and to foster salutogenetic potential. This result confirms the social epidemiological research during the last 20 years, as it shows that social relationships that are experienced as being helpful also promote general well‐being and protect against physical harm (Buchanan 2003). Therefore, health organizations should identify and implement practices that will reduce employee emotional exhaustion and promote social capital in the healthcare organization; it is particularly important to take the necessary precautions to minimize the emotional exhaustion, especially during this pandemic, where the emotional exhaustion level is high. Since it is unclear when this pandemic will completely disappear and whether or not another epidemic will emerge, we must realize the importance of these staff who continue their diligent and devoted work in the field of health.

## Data Availability

Data and materials are available upon request to the corresponding authors.

## Appendix A: items full text

Job demands:

- My pace of work has increased significantly.
  - I’m passing at work more time than I should.
  - The number of patients that I am assisting daily has increased.
  - I’m spending so much energies at work, that my private life has been suffering from it.
  - Since the Covid-19 emergency has begun, I cannot pass enough time with my loved ones.
  - The Covid-19 emergency is forcing me to cope with emotionally difficult situations.
  - The Covid-19 emergency is putting me in touch with other people’s suffering more frequently.
  - The Covid-19 emergency is making me take difficult decisions at work.
  - I often feel like I have to hide my emotions while at work.

Job resources:

- My organization hasn’t adequately trained me on what to do in case of health emergencies such as the Covid-19 (R).
- My organization gave me the Personal Protective Equipment for the management of the Covid-19 emergency.
- I have received all the necessary information from my organization on how to face the Covid-19 emergency.
- My organization is timely in informing me about every important decision regarding the management of the Covid-19 emergency.
- My organization has started initiatives for the psychological support of healthcare workers.
- My organization has developed technological supports to facilitate the continuity of care with patients (for instance, telemedicine…).

Orientation towards patient engagement,

- An active role of the patient in preventing or mitigating the symptoms of Covid-19 is fundamental.
- Regardless of the current situation, I still manage to find alternative ways to maintain a relationship with my patients.
- Regardless of the current situation, I still manage to build a relationship of trust with my patients.
- In the current situation of health emergency, the alliance with the patient is fundamental for the management of care.
- In the current situation of health emergency, it is important that patients and relatives feel authorized to share concerns and questions, even though nobody asked for them.
- In the current situation of health emergency, it is fundamental that patients and relatives are capable of understanding when to get in touch with a healthcare worker and when they can manage their own health themselves.
